# 1388. Epidemiology and Treatment Outcomes of Nontuberculous Mycobacterial Infections at a Community Teaching Hospital in the Southeastern United States

**DOI:** 10.1093/ofid/ofab466.1580

**Published:** 2021-12-04

**Authors:** Y Vivian Tsai, Caroline Derrick, Ismaeel Yunusa, Sharon Weissman, Majdi N Al-hasan, Julie Ann Justo, P Brandon Bookstaver

**Affiliations:** 1 Prisma Health Richland - University of South Carolina, Columbia, South Carolina; 2 University of South Carolina School of Medicine, Columbia, South Carolina; 3 University of South Carolina College of Pharmacy, Columbia, South Carolina; 4 University of South Carolina, Columbia, SC

## Abstract

**Background:**

Gaps in evidence concerning the epidemiology of nontuberculous mycobacterial (NTM) organisms and their associated treatment outcomes are evident in the literature. The aim of this study was to describe NTM species distribution and susceptibility profile and associated treatment outcomes among adult patients at a tertiary referral hospital in the Southeastern United States.

**Methods:**

A retrospective cohort study of adult patients with NTM infections from January 1, 2010 to June 30, 2020 was performed. Included patients had a positive culture for NTM species and clinical suspicion of infection. Patients were excluded if they had concurrent positive culture for *M. tuberculosis* (MTB) or monomicrobial culture for *M. gordonae*. Study endpoints included predictors for favorable treatment outcome, species distribution, and susceptibility at baseline. Favorable treatment outcome was defined as physician-guided cessation of therapy due to clinical improvement. Univariate followed by multivariate regression analysis was used to analyze favorable predictors.

**Results:**

A total of 250 and 78 patients were included in microbiologic and outcomes cohorts, respectively. Among treated patients, 47 (60%) had a favorable treatment outcome. The outcomes cohort consisted primarily of non-Hispanic Caucasians (71%) with pulmonary infection (67%). The most common isolates observed were *Mycobacterium avium complex* (MAC) (67%) and *M. abscessus* (18%). Being self-pay, underweight, history of MTB treatment, and concurrent asthma were more common in those with unfavorable treatment outcomes. The significant favorable predictors included antibiotic change not due to escalation or de-escalation of therapy and private insurance. Among MAC isolates, clarithromycin and amikacin were highly susceptible; however, *M. abscessus* has reduced susceptibility to first-line agents such as amikacin, clarithromycin, and cefoxitin (Table 1).

Table 1. Baseline Susceptibility

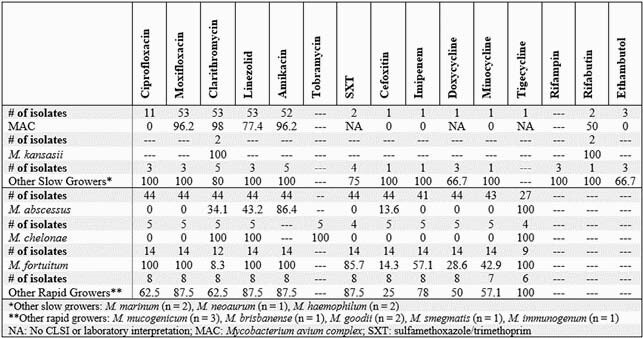

**Conclusion:**

Considering the long incubation time, knowledge of prevalence, antimicrobial susceptibility patterns, and outcomes could guide empirical antimicrobial selection for NTM infections. This is particularly useful for *M. abscessus* infections where most isolates carry significant resistance to one or more first-line agents.

**Disclosures:**

**Julie Ann Justo, PharmD, MS, BCPS-AQ ID**, **bioMerieux** (Speaker’s Bureau)**Merck & Co.** (Advisor or Review Panel member)**Therapeutic Research Center** (Speaker’s Bureau)**Vaxart** (Shareholder) **P. Brandon Bookstaver, Pharm D**, **ALK Abello, Inc.** (Grant/Research Support, Advisor or Review Panel member)**Biomerieux** (Speaker’s Bureau)**Kedrion Biopharma** (Grant/Research Support, Advisor or Review Panel member)

